# Research on two-stage dynamic optimization model and algorithm for agricultural products joint distribution network based on vehicles and customers sharing under data-driven

**DOI:** 10.1371/journal.pone.0323574

**Published:** 2025-06-02

**Authors:** Meilin Zhu, Xiaoye Zhou, Xuan Wang

**Affiliations:** School of management, Shenyang University of Technology, Shenyang, China; SR University, INDIA

## Abstract

Urban road traffic congestion and new customers and customer demands change in the distribution process have brought great challenges to the distribution of agricultural products. Meanwhile, the development of smart logistics makes it possible to share distribution resources, which can provide good help for the efficient distribution of agricultural products. Compared with the static optimization of the distribution network when traditional single enterprise distributes agricultural products, how to dynamically optimize the distribution network of agricultural products under resource sharing has become an urgent problem to be solved. Based on this, this paper takes the joint distribution network of agricultural products as the research object. Firstly, from the perspective of data-driven, it crawls the historical data of driving speed through Baidu map big data platform, and uses a BP neural network optimized by genetic algorithm to predict the driving speed of vehicles in different periods. Secondly, based on the idea of pre-optimization and dynamic adjustment, a two-stage dynamic optimization model of agricultural products joint distribution network under vehicles and customers sharing is established. On this basis, considering the changes of customer demands and the speed of distribution network, a partheno-genetic hybrid simulated annealing algorithm is designed to solve the model by using the idea of disruption event processing combining immediate processing and scheduled batch processing. Finally, the correctness of the model is analyzed through numerical experiments, and the effectiveness of the proposed algorithm, the joint distribution strategy, and the disruption event processing idea of combining immediate processing and scheduled batch processing is analyzed. The research results provide a theoretical basis for agricultural products distribution enterprises to formulate efficient and scientific joint distribution scheme.

## 1 Introduction

In recent years, with China’s policy support for the agricultural products industry chain and the steady increase in market demand for agricultural products, China’s total agricultural products logistics has continued to grow, with a stable growth rate. According to data from the National Development and Reform Commission of China, the total agricultural products logistics in 2022 reached 5.3 trillion-yuan, accounting for 1.52% of the total social logistics, with an annual compound growth rate of 6.66%, which has great development prospects [1]. In addition to facing such a huge demand, agricultural products themselves have the characteristics of short life cycle [2] and perishable [3]. The “last mile” agricultural products distribution in cities also faces the pressure from urban traffic road congestion [4,5], which has brought great challenges to the distribution efficiency and distribution cost of agricultural products. Meanwhile, according to statistics from the China Logistics Information Center, China’s total social logistics costs in 2023 reached 18.2 trillion yuan. This indicator measures the multi-format logistics costs generated by the three major links of transportation, warehousing and management in the process of economic operation from the perspective of the whole society, among which transportation costs account for the highest proportion [6]. Therefore, if the logistics industry wants to achieve cost reduction and efficiency improvement, the optimization of the distribution network is the top priority.

Although China has achieved remarkable results in logistics infrastructure and layout, logistics costs remain high in the face of such huge demand and complex terminal distribution environment. With the development of Internet of Things (IoT) and intelligent transportation systems (ITS) [7,8], it has become possible to collect traffic data from road networks. The changes in traffic flow in these road networks in time and space lead to dynamic fluctuations in the driving speed of distribution vehicles. Compared with other time periods, the congestion index and degree during peak hours are higher than other time periods, and delivery vehicles can only travel at low speeds [9]. Meanwhile, during the distribution process of agricultural products, customers who are unknown in advance will appear and arrive dynamically over time[10–12]. Therefore, in the face of the perishable characteristics of agricultural products and the complex and changeable distribution process at the end of cities, how to integrate a large number of sensors and all kinds of data information obtained by the traffic big data platform in a short time, and reasonably plan the dynamic optimization scheme of agricultural products distribution network under the condition of sharing distribution resources, so as to improve the efficiency of agricultural products distribution at the end of the city, is one of the urgent problems to be solved.

The contributions of this paper are as follows: Firstly, this paper considers two kinds of dynamic disruption events, dynamic traffic network and customer demands change, which can deal with more complex uncertain disruption combinations, expand the existing research, and make it closer to the actual distribution of agricultural products. Considering the time-varying speed of the urban road network, from the perspective of data-driven, this paper uses Python to crawl the historical data of the driving speed of Baidu map big data platform. Meanwhile, considering the nonlinear characteristics of the data, this paper uses BP neural network optimized by genetic algorithm to predict the future driving speed of the road network in different periods. Then, considering the demands of new customers in the distribution process, based on the idea of pre-optimization and dynamic adjustment, a two-stage dynamic optimization model of agricultural products joint distribution network based on vehicles and customers sharing strategy is constructed.

Secondly, to solve the two-stage dynamic optimization model of agricultural products joint distribution network proposed in this paper, a partheno-genetic hybrid simulated annealing algorithm is designed. On one hand, aiming at the disadvantage of premature convergence of genetic algorithm, the simulated annealing mechanism of simulated annealing algorithm is introduced to improve it. The simulated annealing mechanism accepts the poor solution with a certain probability, which not only maintains the global search ability of genetic algorithm itself, but also improves the local search ability of the algorithm. On the other hand, combined with the characteristics of the research problem, the chromosome is encoded with both real numbers and natural numbers, and the optimal segmentation algorithm based on spatio-temporal clustering and Bellman Ford idea is used to decode the chromosome. Finally, single point and multi-point partheno-genetic operations are introduced to improve the global search ability of the algorithm.

Finally, the correctness and effectiveness of the proposed model and algorithm are verified by numerical experiments. In 10 groups of different scale examples, the results of partheno-genetic hybrid simulated annealing algorithm are better than the other two algorithms, and have higher stability. In addition, compared with the traditional single enterprise distribution network of agricultural products, the effectiveness of the vehicles and customers sharing strategy proposed in this paper is proved. Finally, compared with the idea of scheduled batch processing, the superiority of the disruption event processing idea of combining immediate processing and scheduled batch processing in response to new customers is verified. These research results provide a useful reference for the dynamic optimization of the joint distribution network of agricultural products of multiple enterprises.

This paper aims to solve the data-driven dynamic optimization problem of agricultural products joint distribution network. The structure of this paper is divided into six sections. The structure of the paper is as follows: Section 2 reviews the literature on agricultural products distribution network optimization problems, agricultural products distribution dynamic optimization problems, joint distribution problems and data-driven distribution optimization problems, and finds out the research gaps. Section 3 describes the research problem and establishes a vehicle speed prediction model and a two-stage dynamic optimization model for agricultural products joint distribution network optimization based on vehicles and customers sharing. Section 4 designs a partheno-genetic hybrid simulated annealing algorithm to solve the two-stage dynamic optimization model. Section 5 verifies the correctness of the model and the effectiveness of the algorithm through numerical experiments. The last section concludes the paper and outlines potential directions for the future research.

## 2 Literature review

### 2.1 Research on the optimization problem of agricultural products distribution network

In the research field of agricultural products distribution network optimization, the deterministic research assuming that the distribution network environment has complete information and is static and unchanged has been relatively mature, and there are relatively rich research results in various branch systems. Ma et al. [13] took into account the interests of fresh food sellers and outsourced cold chain distribution companies, established a multi-agent optimization model for fresh food distribution location-path based on conflict cooperation, and designed a genetic hybrid particle swarm algorithm to solve model. Govindan et al. [14] studied a two-level location-routing optimization problem, which aims to optimize the distribution cost and carbon emissions by deciding the location and number of distribution centers and optimizing the vehicle delivery routes, and designed an algorithm combining multi-objective particle swarm optimization and adaptive multi-objective neighborhood search to solve it. Taking into account the heterogeneity of demands, Krityakierne et al. [15] designed a hybrid multi-objective evolutionary algorithm to solve the location selection and vehicle route optimization of agricultural products cold chain logistics warehouse facilities. To shorten the overall delivery time of fresh agricultural products, Liu et al. [16] studied the location selection and vehicle distribution path problem of the warehouse center and designed a complex swarm intelligence optimization algorithm based on deep reinforcement learning to solve this problem. Zhou et al. [17] considered the insufficient refrigeration of agricultural products in China’s origin and studied the location selection and path planning of refrigerated warehouses in the origin of fresh agricultural products and designed a hybrid whale optimization algorithm with heuristic rules to solve the LRP model. Chen et al. [18] considered the diverse demands of in-store pickup and delivery customers and established a two-level perishable food distribution network location selection-routing optimization model with the goal of minimizing total cost, and an adaptive large neighborhood search algorithm was developed to solve the model. Wang et al. [19] established a green and low-carbon cold chain logistics location selection-routing optimization model with the goal of minimizing total cost from the perspective of low-carbon environmental protection. Navazi et al. [20] considered the situation of recycling perishable products after spoilage and constructed a closed-loop mathematical model for the location-routing-inventory problem, taking into account factors such as multi-temperature distribution and simultaneous pickup and delivery, and designed the NSGA-II algorithm to solve the model. Wang et al. [21] built a double-objective multi trip vehicle routing optimization model with time windows and considering the order packaging time for fresh products.

### 2.2 Research on dynamic optimization of agricultural products distribution

The research of dynamic optimization of agricultural products distribution network belongs to the category of dynamic location routing problem (DLRP) of logistics distribution network, which can be traced back to 1988 [22]. Compared with the static optimization problem of agricultural products distribution network, the essential difference between the two lies in the real-time and dynamic nature of information. In the dynamic optimization problem of agricultural products distribution network, part of the information of the problem is gradually disclosed or changed during the execution of the distribution plan, which leads to the continuous adjustment of the distribution network optimization plan. There are three main types of dynamic factors considered in the existing research on dynamic optimization of agricultural products distribution network:

(1) Dynamic traffic network, such as road disruption or traffic congestion. Luo et al. [23] considered the impact of traffic and weather conditions on vehicle speed, established the driving speed function under the influence of traffic and weather conditions, and designed an improved ant colony algorithm to solve the model. Liu et al. [24] took into account both economic and environmental costs and studied the vehicle routing problem with time windows for fresh food e-commerce distribution under time-varying road networks. Fu et al. [25] considered the perishability of agricultural products and introduced the freshness measurement function of agricultural products under normal temperature conditions to study the open time-varying vehicle routing problem with freshness constraints.
(2) Changes in customer demands mainly include five types: demands from new customers, changes in the time window demands of original customers, cancellation of original customer demands, reduction in original customer demands, and increase in original customer demands. After the distribution center receives real-time customer demands information, the basic processing idea is to decompose the complex dynamic problem into several static sub-problems. For the situation of new customers or changes in the demands of existing customers, scholars usually adopt the following two processing ideas [26,27]: ① immediate processing; ② batch processing.
(3) Vehicle conditions, such as vehicle failure. Vehicle failure is a common random disruption event in agricultural products distribution. It refers to the situation in which a distribution vehicle cannot continue, has to delay or interrupt the distribution task due to a mechanical failure during the distribution process. There are two types of vehicle failure events: one is repairable, and the vehicle can continue to perform the task after repairing, the other is irreparable within the allowed time range, and the faulty vehicle has to interrupt the distribution task. Existing research mainly focuses on the second situation, that is, when the vehicle fails and cannot be recovered, the distribution center usually adopts the following two rescue methods: ① rescued by neighboring vehicles on the way. ② rescued by additional vehicles.

Ding et al. [28] introduced the idea of disruption management into the real-time dynamic distribution of agricultural products, measured the disruption degree by the freshness of agricultural products and the distribution service time, created a disruption recovery model for vehicle failure in the cold chain logistics distribution of agricultural products, and verified the advantages of disruption management in dealing with vehicle failure disruption. Ding et al. [29] combined the research methods of behavioral perception in behavioral science and quantitative optimization in operations research, analyzed the impact of disruption events on the three main bodies of producers, customers and logistics distribution operators, and created a disruption management model for the cold chain logistics distribution of fresh agricultural products.

### 2.3 Research on joint distribution

With the rapid development of the IoT, artificial intelligence technologies and the deepening of the construction of shared information platforms, intelligent and shared agricultural products distribution networks have become a trend [30,31]. In particular, considering the perishable characteristics of agricultural products, it is difficult for enterprises to meet their timeliness requirements by independently carrying out distribution tasks. Scholars have considered introducing the sharing of different distribution resources among multiple entities into the research on agricultural products distribution, mainly involving distribution centers, vehicles, and customers [32,33]. Zhang et al. [34] took the “contactless” distribution of fresh goods under the epidemic environment as the research background and proposed the problem of drone site selection and vehicle-drone collaborative distribution path optimization. Liu et al. [35] studied the dairy vehicle routing problem considering the rental of shared vehicles in view of the company’s own insufficient transportation capacity, and designed a genetic algorithm combining scanning algorithm and saving algorithm. Wang et al. [36] proposed the vehicle routing optimization problem of multi-center joint distribution of fresh products with integrated vehicle sharing and temperature control in view of the perishable characteristics and untimely distribution in fresh products distribution, and designed the NSGA-II algorithm combined with tabu search to solve it. Xia et al. [37] studied the fresh products vehicle routing optimization problem with splitable demands considering customer grading and customer resource sharing, and developed a tabu search algorithm with a dynamic tabu table to solve it. It can be seen from the above literature that sharing of distribution resources can provide good help in improving distribution efficiency and reducing distribution costs, and provide a research reference for the agricultural products joint distribution network optimization problem studied in this paper.

### 2.4 Research on data-driven distribution optimization

In recent years, many researchers have optimized management decision-making problems from a data-driven perspective by analyzing relevant data mining rules and combining data analysis results [38], such as data-driven shared travel resource allocation problems [39] and data-driven takeaway delivery path optimization problems [40]. Li et al. [41] considered the uncertainty of AGV travel time at automated terminals and proposed a data-driven two-layer distributed robust optimization model of quay cranes and AGVs. Du et al. [42] considered the uncertainty of infectious disease transmission parameters and epidemic status, and combined with the characteristics of periodic arrival of confirmed data, proposed a data-driven online decision-making method that integrates stochastic programming and rolling optimization to achieve accurate prediction of epidemic transmission trends and reasonable allocation of epidemic emergency resources. Sarkar et al. [43] proposed a data-driven hospital patients allocation model, which includes a new coronavirus infectious disease model and a patient allocation model. Chu et al. [44] considered the uncertainty of travel time and proposed to use a linear regression model to predict delivery time and then optimize the delivery path. However, research on the optimization of agricultural products distribution network from a data-driven perspective is relatively rare. Ma et al. [45] addressed the problem of optimizing the path of fresh food distribution vehicles with varying vehicle speeds under a time-varying road network, and used an LSTM model to predict the actual vehicle speed based on historical driving speed data, and then designed an adaptive genetic algorithm to solve the optimization model under a time-varying road network, which is closer to the actual distribution situation.

### 2.5 Research gaps

In reality, the distribution of agricultural products is affected by the changes of customer demands and the time-varying speed of urban road network, and the distribution efficiency of agricultural products of a single enterprise and the utilization efficiency of resources such as vehicles are often low. However, the existing research does not fully consider these practical factors in the distribution of agricultural products. Table 1 introduces the contributions and features of this study.

**Table 1:** Abstracts of relevant literature.

Author	Time-dependent	Newly arriving customers	Resources sharing	Multi-depot	freshness	Immediate processing and scheduled batch processing
	With/Without	With/Without	With/Without	With/Without	With/Without	With/Without
Ma et al. [13]		√				√
Govindan et al. [14]				√	√	
Luo et al. [23]	√				√	
Liu et al. [24]	√				√	
Li et al. [26]	√	√			√	
Fan et al. [27]	√	√				√
Zhang et al. [34]			√		√	
Xia et al. [37]			√		√	
Ma et al. [45]	√				√	
Wang et al. [33]			√	√	√	
This paper	√	√	√	√	√	√

For the lack of previous research, this paper starts from the data-driven perspective, and comprehensively considers the processing ideas of disruption events such as time-varying vehicle speed, new customers in the process of distribution, distribution resources sharing, multiple distribution centers, freshness, and the combination of immediate processing and scheduled batch processing in the construction of the two-stage dynamic optimization model of agricultural products joint distribution network, which effectively reduces the total cost of agricultural products distribution, improves the efficiency of agricultural products distribution, and timely responds to new customers in the process of distribution.

## 3 Problem description and model formulation

### 3.1 Formulated problem

**Fig 1:**
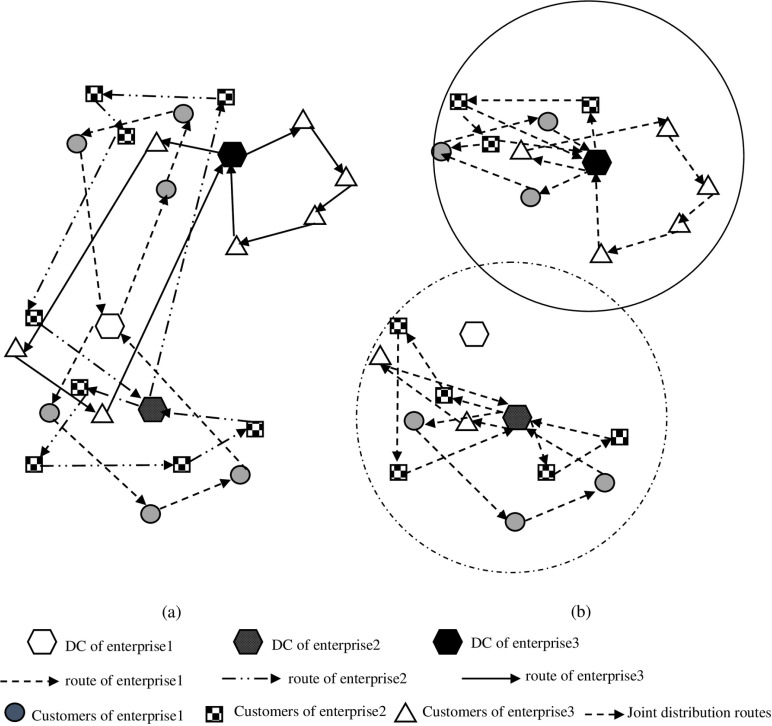
Schematic diagram of the comparison of the agricultural product joint distribution network before and after optimization under the sharing of vehicles and customers.

The logistics demands of agricultural products at the end of the city presents the characteristics of small batch, high frequency and dynamic. In addition, the perishable characteristics of agricultural products themselves make logistics enterprises face problems such as high cost, distribution delay and long-distance distribution when carrying out distribution services independently, as shown in Fig 1(a), which is easy to cause serious damage to the agricultural products delivered to customers. In order to achieve cost reduction and efficiency increase in the “last mile” of urban agricultural products distribution, this paper proposes a joint distribution network of agricultural products based on vehicles and customers sharing, as shown in Fig 1(b). There are several alternative distribution centers and customers with known geographical location, demands and time window. Each alternative distribution center is equipped with a sufficient number of vehicles to complete the distribution of agricultural products. To improve the distribution efficiency, considering the customer service time window, demands, vehicle capacity and other factors, which alternative distribution centers should be opened. On the premise that each customer can only be served by one vehicle once, reasonably dispatch vehicles to deliver agricultural products to customers, and return to the distribution center at the time of departure after the distribution task.

The distribution process is divided into two stages. The first stage is the pre-optimization stage, which carries out the initial optimization modeling combined with the location of the alternative distribution centers, the known location and demands of the initial customers booked the previous day, and selects the appropriate distribution center, and designs the initial vehicle distribution paths. The initial customers refer to the customers who submit the demands before the distribution center starts working. However, within a certain period of time when the distribution vehicles perform the distribution task according to the initial scheme, there may be new customer demands, cancellation, increase or decrease of the initial customer demands. At this time, it will enter the second stage of dynamic adjustment. The selected distribution center will set the location of the vehicles in transit as virtual nodes according to the real-time information collected by the IoT, and adjust the distribution paths and vehicle configuration of subsequent unserved customers.

The objective function of the first stage model minimizes the total cost including operation cost of selected distribution center, vehicle fixed cost, vehicle driving cost, agricultural products loss cost and penalty cost caused by violating the time window. The objective function of the second stage model includes vehicle fixed cost, vehicle driving cost, agricultural products loss cost and penalty cost caused by violating the time window.

The main decisions include: ①Selection of distribution centers. ②Reallocation of service customers to the selected distribution center. ③Service routing of the vehicles.

Considering the actual situation that the vehicle speed is time-varying in the process of agricultural products distribution, and without losing generality, the following assumptions are put forward for the model: ①There is a complete Internet of things environment. ②After the vehicle departs from the distribution center to visit the customers who need services, it needs to return to the departing distribution center. ③All delivery vehicles are homogeneous. ④The customer’s demands for agricultural products are known. ⑤Each customer can only be served once. ⑥The distribution center has enough capacity to meet the demands of customers.

**Fig 2:**
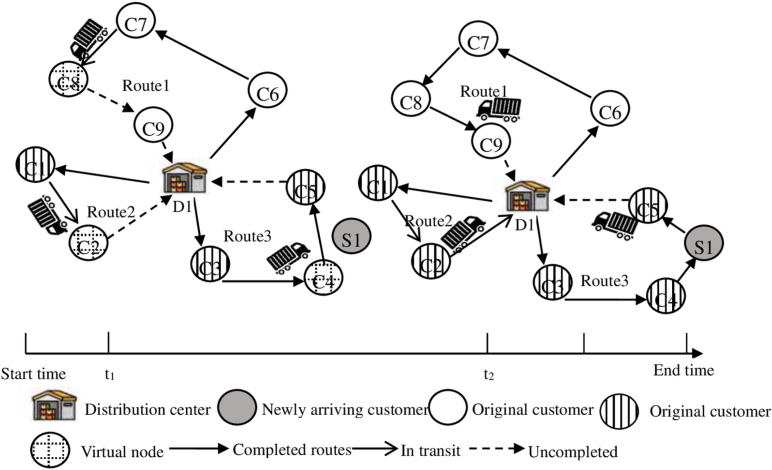
Key nodes and adjusting vehicle routings.

Taking Fig 2 as an example, the routing adjustment in the dynamic adjustment stage is explained.

As shown in Fig 2, according to the known customers’ information (C1-C9) and road network conditions, an initial optimization scheme for the joint distribution network of agricultural products that minimizes the total cost of distribution is formulated, taking the new customers in distribution center D1 as an example. Record that the time when the new customer appears is *t1*. At *t1*, the new customer S1 appears when each vehicle is performing the distribution task according to the initial distribution scheme. At this time, the customer C4 location where *vehicle 3* is located and the C8 and C2 locations where *vehicles 1* and *2* are about to arrive are used as virtual nodes. The distribution center adjusts the number of vehicles and the corresponding distribution routes according to the real-time information, including the new customer S1 in the customer set, and adjust the vehicle distribution routes. If the remaining load capacity of the vehicle in transit cannot meet the demands of all remaining undelivered customers, a new *vehicle 4* shall be dispatched. At *t2*, it shows that the delivery of the new customer S1 has been completed.

### 3.2 Explanation of symbols

The symbols and meanings of sets, parameters and decision variables involved in the data-driven two-stage dynamic optimization model of agricultural products joint distribution network based on vehicles and customers sharing are shown in Table 2.

**Table 2:** Explanation of symbols and meanings.

Symbols		Description
Set	R	Set of alternative joint distribution centers, ∀r∈R
	$N_{1}$	Set of initial customers, $\forall i,j\in N_{1}$
	$N_{2}$	Set of dynamic customers, $\forall g,h\in N_{2}$
	K	Set of vehicles, ∀k∈K
Parameter	$F_{R}$	Operating costs of distribution centers
	$F_{K}$	Fixed cost of starting a vehicle once
	$F_{d}$	Vehicle’s driving cost per unit time (unit: yuan/hour)
	$F_{S}$	Unit cost of agricultural products damage
	$\varphi_{1}$	Waiting penalty cost per unit time
	$\varphi_{2}$	Cost of penalty for lateness per unit time
	$Q_{K}$	Capacity of delivery vehicles
	$d_{ij}$	The distance between node i and node j
	$q_{i}$	The agricultural products demand quantities of the customer i
	$T_{S}$	Start working time of distribution center
	$T_{E}$	End working time of distribution center
	$s_{i}$	Service time of customer i
	$U_{ik}$	The in-transit load of vehicle k after leaving customer i
	$ET_{i}$	The earliest time when agricultural products are delivered to customer i
	$LT_{i}$	The latest time when agricultural products are delivered to customer i
	$\beta_{1}$	The damage coefficient of agricultural products during distribution
	$\beta_{2}$	Damage coefficient of agricultural products during loading and unloading
	$\left|S_{k}\right|$	The number of customers served by vehicle k
	$t_{ijk}$	The time required for vehicle k to travel between (i,j)
Decision variables	$X_{r}$	Equal to 1 if and only if the alternative distribution center r is selected for joint distribution
	$Y_{ir}$	Equals 1 if and only if the initial customer’s agricultural products demands is provided by the selected distribution center r
	$Z_{ijk}$	It is equal to 1 if and only if the vehicle k passes by (i,j)
	$T_{ik}$	The time when the delivery vehicle k arrives at the customer i

### 3.3 Model formulation

**Fig 3:**
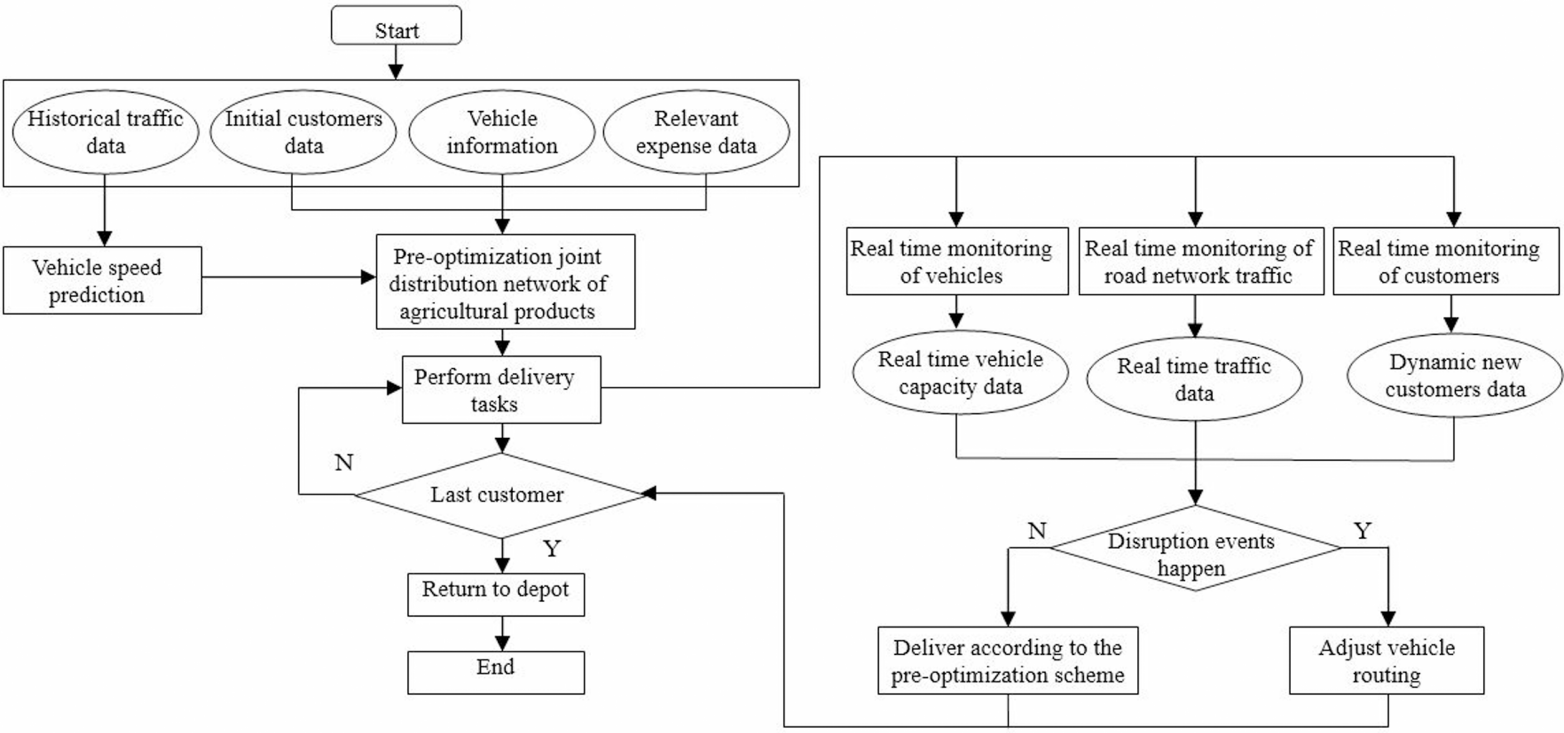
Two-stage dynamic optimization process of agricultural products joint distribution network based on data driven under IoT.

Addressing the issues of low efficiency, information lag, and high costs faced by traditional agricultural products distribution, this paper establishes a two-stage dynamic optimization model for an agricultural products joint distribution network based on vehicles and customers sharing, supported by IoT technology. To more clearly illustrate how real-time tracking by IoT affects the real-time response capability of the agricultural products joint distribution network, this paper depicts the two-stage dynamic optimization process of the IoT-based data-driven agricultural products joint distribution network, as shown in Fig 3.

The specific process is as follows:

Step1: Based on historical speed data from the urban road network, the GA-BP neural network is employed to predict future driving speeds. This predicted speeds, combined with data from initial customers (those who submit their demands before the distribution center begins operations), vehicle information, relevant cost data, and other relevant data, generates an initial optimization plan for the joint distribution network of agricultural products.
Step2: Vehicles execute distribution tasks according to the optimized plan, while IoT conducts real-time monitoring and information collection for vehicles, customers, and traffic using technologies such as RFID, GPS, and GIS.
Step3: If disruption events occur during the distribution process, such as new customers or changes in original customer demands, the distribution center adjusts the delivery sequence and vehicle configuration for subsequent unserved customers based on real-time information. Vehicles in transit complete their deliveries according to the adjusted routes, and determine whether the last customer has been delivered. If so, proceed to step 4. Otherwise, return to step 2.
Step4: Return to the depot, complete all delivery tasks.

#### 3.3.1 Road network data acquisition and data-driven road network travel speed prediction.

In recent years, the contradiction between the growing number of vehicles and the limited road resources has become increasingly prominent, and the resulting road congestion problem has a great impact on people’s daily commuting. With the development of intelligent transportation technology and big data technology, it is possible to accurately predict the future traffic flow and traffic congestion index, so as to deal with the time-varying speed and help travelers make reasonable travel plans.

**Fig 4:**
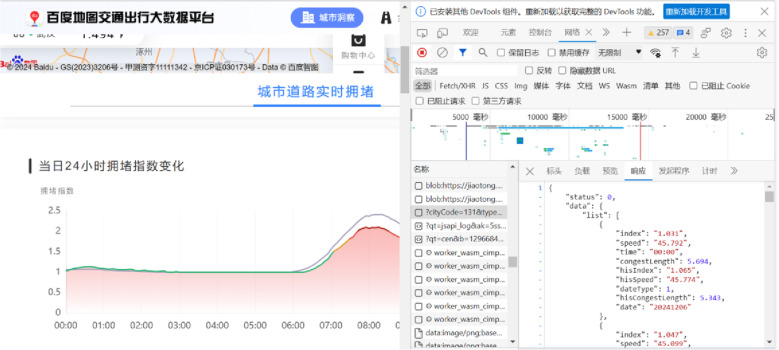
City driving speed query results.

This paper uses Python to initiate a request to the Baidu Map Big Data Platform through the API, obtain the standard data stream returned in JSON format, and then parse, process, store and perform a series of subsequent operations on the data stream. The Baidu Map Big Data Platform API provides data interaction functions based on the browser/server mode (Browser-Server, B/S) of the HTTP/HTTP protocol. It needs to simulate the browser to send requests to the server, and then judge whether the query is successful based on the JSON data returned by the Baidu Map server, and finally extract useful information from the JSON data string. The basic process of API call in Baidu Map Big Data Platform is as follows: first, apply for an account and enter the console of the developer platform to apply for personal developer certification. Second, create the corresponding service key (Activation Key, AK) according to different application types, and start the API call service. Finally, set parameters and initiate requests through HTTP/HTTPS and other interfaces to extract the required information from the returned JSON or XML format data. Taking the historical speed of the city as an example, the browser can be simulated to send the following GET request “https://jiaotong.baidu.com/trafficindex/city/curve/?cityCode=131&type=minute&his=1&from=” to Baidu Map. After the query is issued, the JSON string of Baidu Map response is structured, as shown in Fig 4.

Whereas, status: 0 indicates that the query is successful. At this time, the city traffic congestion index can be extracted from the index field of the list, and the driving speed at the corresponding time can be extracted from the speed field. According to the Baidu Big Data Traffic Platform, updating data every 5 minutes, the working time window of the distribution center is divided into several time periods of 5 minutes in length, and it is assumed that the severe congestion period in a period of traffic congestion is continuous, and the driving speed in the same period will not change.

The obtained driving speed data set needs to be preprocessed before being used for prediction. The data preprocessing techniques used in this paper mainly include: analysis and processing of outliers and missing values, and sliding window setting. Among them, the sliding window is a commonly used data enhancement method. To make full use of the information contained in the historical data to predict the target value, this paper reshapes the original data set and then performs the sliding window operation.

Considering that the collected road network speed data changes continuously at intervals of 5 minutes and has typical nonlinear characteristics, this paper adopts BP neural network to predict the road network speed in different time periods in the future, and uses genetic algorithm to optimize the BP neural network parameters. Suppose the predicted value of the road network speed at time t is v(t), $v_{d}\left(t\right)$ represents the road network speed at time t on the same day last week, $v_{b}\left(t\right)$ represents the road network speed at time t the day before yesterday, $v_{y}\left(t\right)$ represents the road network speed at time t yesterday, v(t−T), …, v(t−nT), represents the road network speed in the n time periods before this, where T represents the time interval. Since the road network speed in the Baidu Map Big Data Platform is updated every 5 minutes, the number of time periods is set to n=5, so the number of neurons in the input layer of the BP neural network is 8, and the number of neurons in the output layer is 1.

#### 3.3.2 Pre-optimization stage model.

- (1) Agricultural products loss cost

The freshness of agricultural products will decrease over time. The loss function used in this paper is shown in Eq (1).


$$\lambda_{1}=\lambda_{0}\exp\left(-\beta t\right)$$ (1)

Eq (1) is an exponential loss function, where $\lambda_{0}$ represents the mass constant of fresh agricultural products when they leave the distribution center, $\lambda_{0}=1$. β represents the spoilage coefficient of agricultural products. The agricultural products loss costs considered in this paper include two parts: the loss costs during the distribution process and the damage costs during the loading and unloading process. Then the agricultural products loss cost AC can be expressed as Eq (2).


$$AC=F_{S}\sum_{i\in R⋃N_{1}}\sum_{j\in N_{1}}\sum_{k\in K}Z_{ijk}U_{ik}\left(1-e^{-\beta_{1}(T_{ik}-T_{S})}\right)+F_{S}\sum_{i\in N_{1}}\sum_{k\in K}Z_{ijk}U_{\left(i-1\right)k}\left(1-e^{-\beta_{2}s_{i}}\right)$$ (2)

- (2) Mathematical model

The pre-optimization stage model aims to minimize the total distribution cost, including the operating cost of the distribution center, the vehicle fixed cost, the vehicle driving cost, the penalty cost caused by violating the time window, and the agricultural products loss cost.


minTC=DC+VVC+CVC+PC+AC (3)

$$DC=\sum_{r\in R}F_{R}X_{r}$$ (4)

$$VVC=\sum_{r\in R}\sum_{i\in N_{1}}\sum_{k\in K}F_{K}x_{ijk}$$ (5)

$$CVC=\sum_{i\in N_{1}}\sum_{j\in N_{1}}\sum_{k\in K}F_{d}t_{ijk}x_{ijk}$$ (6)

$$PC=\sum_{i\in N_{1}}\sum_{j\in N_{1}}\max\left[\varphi_{1}\left(ET_{i}-T_{ik}\right)Z_{ijk},0\right]+\sum_{i\in N_{1}}\sum_{j\in N_{1}}\max\left[\varphi_{2}\left(T_{ik}-LT_{i}\right)Z_{ijk},0\right]$$ (7)

Constraints:


$$\sum_{i\in N_{1}}\sum_{k\in K}q_{i}Z_{ijk}\leq Q_{k},\forall k\in K$$ (8)

$$\sum_{i\in N_{1}}\sum_{j\in N_{1}}Z_{ijk}=\left|s_{k}\right|-1,\forall k\in K$$ (9)

$$\sum_{i\in R⋃N_{1}}Z_{ijk}-\sum_{i\in R⋃N_{1}}Z_{jik}=0,\forall k\in K,j\in N_{1}$$ (10)

$$\sum_{i\in N_{1}}\sum_{j\in N_{1}}Z_{ijk}=\sum_{j\in N_{1}}\sum_{i\in N_{1}}Z_{jik}=1,\forall k\in K$$ (11)

$$T_{jk}=T_{ik}+t_{ijk}+s_{i},\forall i\in N_{1}⋃R,k\in K$$ (12)

In Eq (3), DC is the operating cost of distribution center, VVC is the vehicle fixed cost, CVC is the vehicle driving cost, PC is the penalty cost caused by violating the time window, and AC is the agricultural products loss cost. Eq (8) to (13) are constraint conditions. Eq (8) represents the vehicle loading capacity. Eq (9) ensures that there are no sub-loops in the path. Eq (10) indicates that the vehicle needs to leave after serving the customer to ensure the balance of inflow and outflow. Eq (11) ensures that there is only one vehicle serving each customer. Eq (12) represents the relationship between the arrival time of vehicles between adjacent nodes in one routing.

#### 3.3.3 Dynamic adjustment ideas and dynamic adjustment stage model.

- (1) Dynamic adjustment ideas

After the distribution center receives the real-time customer demand information, the basic processing idea is to decompose the complex dynamic problem into several static subproblems. For new customers or changes in the demands of original customers, this paper adopts the following two treatment ideas: ① Immediate processing. ② Scheduled batch processing.

The idea of immediate processing means that when the distribution center receives real-time customer information, it will update and adjust the number of vehicle configurations and their driving routes in real time considering the unserved customer information, vehicle information and changes in the demands of new customers or original customers. The distribution center can immediately respond to the real-time information.

The idea of scheduled batch processing is to divide the working time window of the distribution center into several equal time periods, and the new customers and unserved customers in each time period are static. The idea of scheduled batch processing can avoid frequent changes in the path scheme due to the frequent occurrence of new customers.

- (2) Dynamic adjustment stage model

The variables involved in the dynamic adjustment stage model are explained as follows: the time when the new customer appears is $T_{N}$, $t_{n}$ is the end time of the nth period, $M_{n}$ is the set of new customers and the original unserved customers $M_{N_{1}}$, $K^{\prime }$ is the set of vehicles in transit in the distribution network, $Q_{k^{\prime }}$ is the remaining amount of agricultural products in the vehicles in transit, $K^{\prime \prime }$ is the set of new vehicles that can be dispatched by the distribution center, $K^{\prime \prime \prime }=K^{\prime }\cup K^{\prime \prime }$.

The goal of the dynamic adjustment stage model is to minimize the sum of costs including vehicle fixed costs, vehicle driving costs, penalty costs caused by violating the time window, and agricultural products loss costs.


$$\begin{array}{c} \min\sum_{j\in M_{n}}\sum_{k\in K^{\prime \prime \prime }}F_{K}Z_{rjk}+\sum_{i\in R^{\prime }\cup M_{n}}\sum_{j\in R^{\prime }\cup M_{n}}\sum_{k\in K^{\prime \prime \prime }}F_{d}t_{ijk}Z_{ijk}\\ \text{+ }\sum_{i\in R^{\prime }\cup M_{n}}\sum_{j\in M_{n}}\sum_{k\in K^{\prime \prime \prime }}Z_{ijk}F_{S}U_{ik}\left(1-e^{-\beta_{1}(T_{ik}-T_{S})}\right)+\sum_{i\in M_{n}}\sum_{k\in K^{\prime \prime \prime }}Z_{ijk}F_{S}U_{\left(i-1\right)k}\left(1-e^{-\beta_{2}s_{i}}\right)\\ \text{+}\sum_{i\in M_{n}}\sum_{j\in M_{n}}\max\left[\varphi_{1}\left(ET_{i}-T_{ik}\right)Z_{ijk},0\right]+\sum_{i\in M_{n}}\sum_{j\in M_{n}}\max\left[\varphi_{2}\left(T_{ik}-LT_{i}\right)Z_{ijk},0\right]\; \end{array}$$ (13)

$$\sum_{i\in R^{\prime }⋃M_{n}}\sum_{j\in M_{n}}Z_{ijk^{\prime }}q_{i}\leq Q_{k^{\prime }},\forall k^{\prime }\in K^{\prime }$$ (14)

$$\sum_{i\in R^{\prime }⋃M_{n}}\sum_{j\in M_{n}}Z_{ijk^{\prime \prime }}q_{i}\leq Q_{k},\forall k^{\prime \prime }\in K^{\prime \prime }$$ (15)

$$\sum_{i\in R^{\prime }\cup M_{n}\cup vc}\sum_{k\in K^{\prime \prime \prime }}Z_{ijk}=1,\forall j\in M_{n}$$ (16)

$$\sum_{j\in R^{\prime }\cup M_{n}\cup vc}\sum_{k\in K^{\prime \prime \prime }}Z_{jik}=1,\forall i\in M_{n}$$ (17)

$$\sum_{k\in K^{\prime }}\sum_{j\in M_{n}}Z_{ijk}=1,\;\forall i\in vc$$ (18)

$$\sum_{k\in K^{\prime }}\sum_{i\in R^{\prime }⋃M_{n}}Z_{ijk}=1,\;\forall j\in vc$$ (19)

The objective function Eq (13) represents the minimization of the distribution cost. The first part is the vehicle fixed cost, the second part is the vehicle driving cost, the third part is the agricultural products loss cost, and the fourth part is the penalty cost caused by not meeting the customer’s time window. Eqs (14) and (15) constrain the loads limit of vehicles in transit and newly dispatched vehicles. Eqs (16) and (17) indicate that a customer is only served once by one vehicle. Eqs (18) and (19) restrict vehicles from returning to a virtual node after leaving it, and only one vehicle leaves each virtual node.

## 4 Algorithm design of two-stage dynamic optimization model for agricultural products joint distribution network

As we all know, LRP is a NP-hard problem. As an extension of LRP, the dynamic optimization of agricultural products joint distribution network studied in this paper is also a NP-hard problem. It is difficult to find a satisfactory solution by accurate algorithm. In recent years, genetic algorithm has excellent performance in solving NP-hard problem. Because of its good robustness and global search performance, it is widely used to solve the distribution network optimization problem. However, the algorithm is prone to premature convergence. In view of the shortcomings of genetic algorithm, this paper proposes a partheno-genetic hybrid simulated annealing algorithm (PGHSAA) by combining partheno-genetic algorithm with simulated annealing algorithm. In PGHSAA, metropolis mechanism of simulated annealing algorithm is introduced to improve it. The simulated annealing mechanism accepts the poor solution with a certain probability, which not only maintains the global search ability of the genetic algorithm itself, but also improves the local search ability of the algorithm.

**Fig 5:**
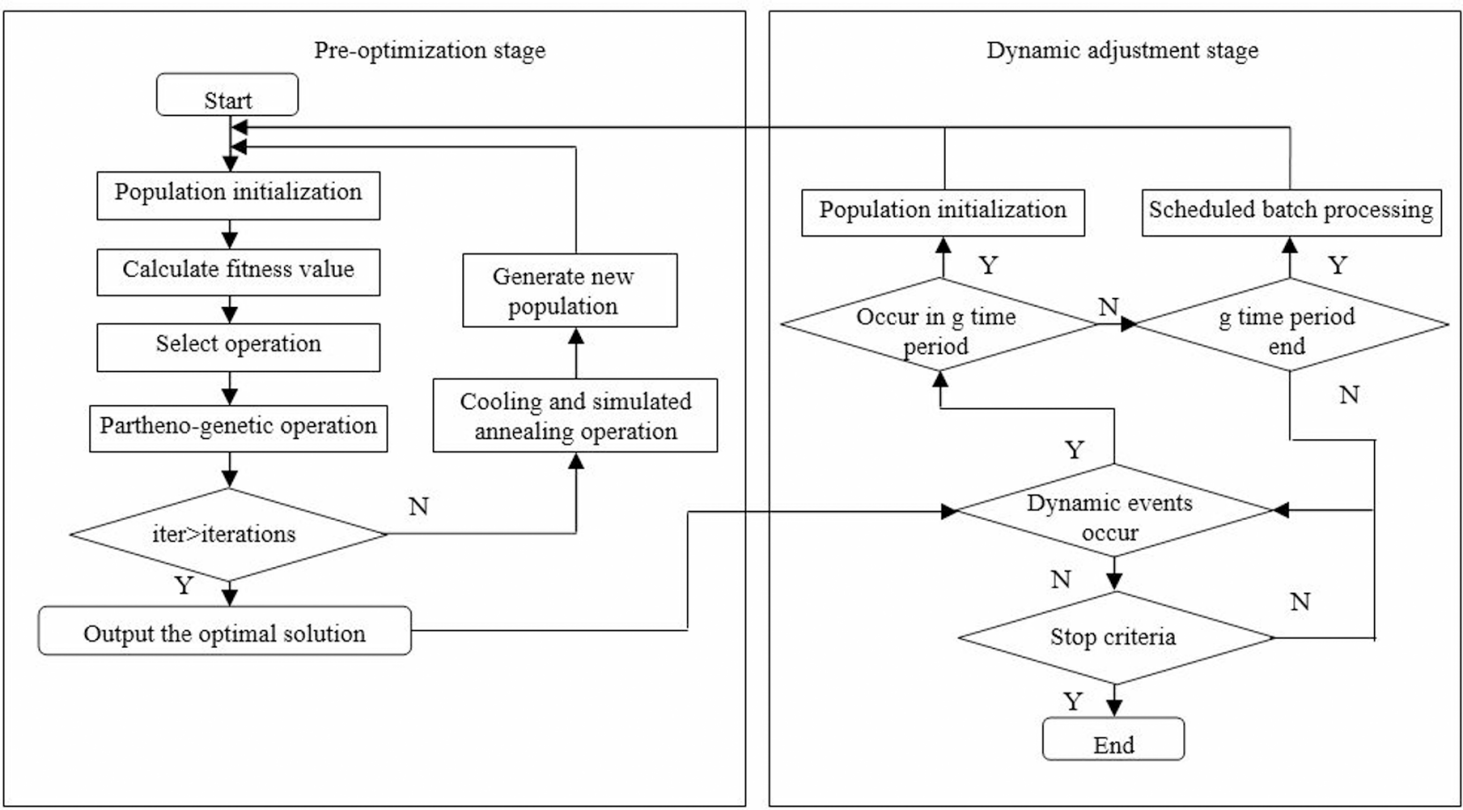
The flow chart of PGHSAA.

In the pre-optimization stage, the site selection of the distribution center and the vehicle route optimization are completed. The site selection of the distribution center and the distribution route arrangement are completed by combining the location of the alternative distribution center, customer coordinates, time windows and demands, and vehicle information. The model is solved using the PGHSAA to obtain the initial optimization plan for the joint distribution network of agricultural products. In the dynamic adjustment stage, the dynamic route adjustment is completed. During the vehicle distribution process, if new customer demands appear or the original customer demands change, it is necessary to combine the planned initial optimization plan, real-time vehicle location, remaining load capacity, and information of customers that have not yet been delivered, and use the dynamic adjustment stage algorithm to adjust the distribution route in time, the flow chart of PGHSAA for solving the two-stage dynamic optimization model of agricultural products joint distribution network is shown in Fig 5.

### 4.1 Algorithm design in the pre-optimization stage

**Fig 6:**
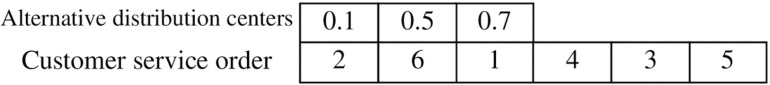
Schematic diagram of chromosome coding.

Firstly, on the basis of the traditional genetic algorithm framework, combined with the characteristics of the research problem, the mixed real number and natural number coding mechanism, and the optimal segmentation algorithm decoding mechanism based on spatio-temporal clustering and Bellman Ford idea are designed. Secondly, single point and multi-point partheno-genetic operations are introduced to improve the global search ability of the algorithm. Finally, the simulated annealing algorithm is introduced into the evolution process of genetic algorithm to accept the poor solution with a certain probability, which ensures the local search ability of genetic algorithm. The specific flow of the algorithm is as follows:

Step 1: Chromosome encoding and decoding. The chromosomes in this paper are divided into two parts: the chromosomes of the candidate distribution center location selection part are encoded with real numbers, and the chromosomes of the customer service order part are encoded with natural numbers. Take Fig 6 as an example to explain the chromosome encoding in detail. Fig 6 contains 3 candidate distribution centers and 6 customers.

**Fig 7:**
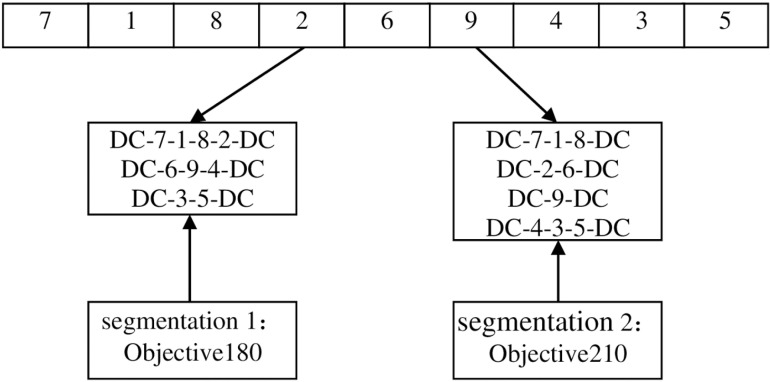
Initialize the delivery segmentation map.

In the first part, the variable of whether each candidate distribution center is selected is set to [0,1]. The location variables in the first part are rounded to get the preliminary location plan. If the genes after rounding in the first part are all 0, it means that no distribution center is selected in the preliminary location selection, and the alternative distribution center 1 is selected. On this basis, the spatiotemporal clustering algorithm [46] is used to determine the customers that the selected distribution center is responsible for, taking into account time and space factors. On this basis, different vehicles are arranged to serve customers, the Bellman-Ford idea is introduced, and the optimal segmentation algorithm is used to realize vehicle service path decoding. The central idea of the optimal segmentation is to set each gene position in the chromosome as a point. Assuming that the road section (i,j) is connected and its path satisfies the vehicle load constraint and the time window constraint, it is considered that there is an edge with a weight of the path length between (i,j). The optimal segmentation is the shortest path from the first gene point of the chromosome to the last gene point, that is, the path with the smallest objective function. The initial distribution segmentation plan containing 9 customers is shown in Fig 7. The objective function of segmentation method 1 is smaller, so segmentation method 1 is selected.

Step2: Initialize the population. As a group search algorithm, the initial population is the search starting point of the partheno-genetic algorithm, which is generally randomly selected. The reason is that theoretically, only under random selection can the evolution of the population traverse all states and find the global optimal solution. The selection based on a specific rule will reduce the diversity of the population and cause the population to converge prematurely. Therefore, this paper adopts a random generation method to obtain the initial population.

Step3: Calculate the fitness function. The fitness function is a main indicator used to measure the adaptability of an individual. Generally, the larger the fitness value of an individual, the greater the probability that it will be inherited to the next generation of the population. Eliminate individuals with low fitness, select individuals with high fitness as the next generation of the population, and inherit their excellent genes to the next generation. The objective function of the model established in this paper is to minimize the distribution cost, and the inverse of the objective function is constructed as the fitness function, that is, the smaller the objective function value, the larger the fitness value. The larger the objective value, the smaller the fitness value.

Step4: Selection operator based on roulette. All individuals in the population are selected according to the “roulette wheel” method to generate a new population. The top 20% of the best individuals in the original population are merged with the top 80% of the best individuals in the new population to participate in the subsequent partheno-genetic operation.

Step 5: Partheno-genetic operation. All genetic operators of partheno-genetic operation are only performed on one parent chromosome, and it has been verified that the optimization efficiency and overcoming “premature convergence” are better than traditional genetic algorithms [47]. This paper adopts three partheno-genetic operators: gene transposition, gene shift and gene inversion. All genetic operations can be divided into single-point and multi-point gene operations. Given [aa,bb], a random number *rand* is given. If *rand* is less than aa, gene transposition is performed. If it is greater than bb, gene inversion is performed, otherwise gene shift operation is performed.

(1) Gene transposition operator

Gene transposition operators include single-point gene transposition and multi-point gene transposition. The gene transposition operator is to exchange genes at any two (or some) positions on the chromosome with a certain probability.

**Table:** 

Single-point gene transposition:	Multi-point gene transposition:
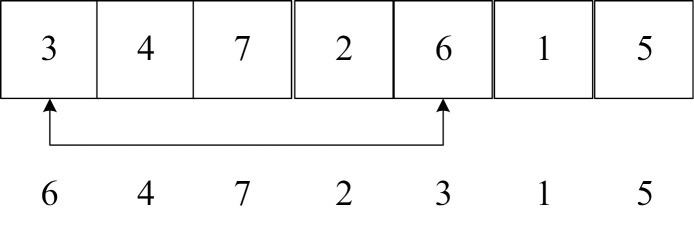	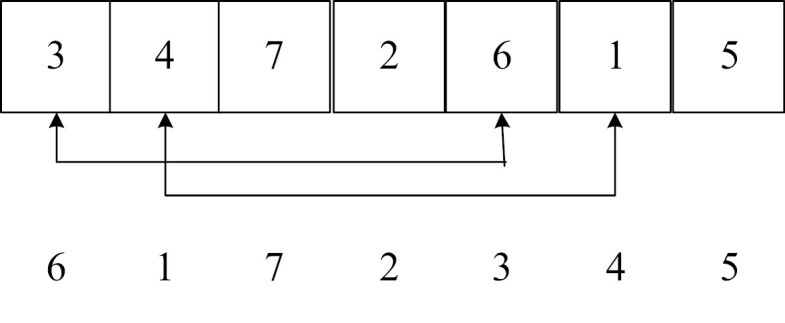

(2) Gene shift operator

Gene shift operators include single-point gene shift and multi-point gene shift. The gene shift operator is to intercept any substring (or substrings) of the chromosome and transfer the last gene in the substring to the first gene in the substring.

**Table:** 

Single-point gene shift:	Multi-point gene shift:
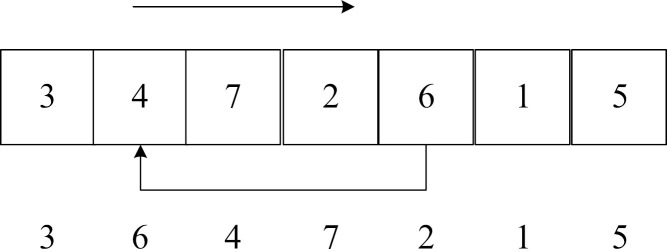	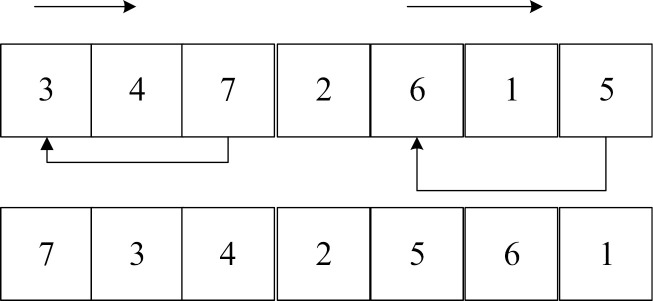

(3) Gene inversion operator

Gene inversion operators include single-point gene inversion operators and multi-point gene inversion operators. The gene inversion operator first randomly cuts a substring from the parent chromosome and reverses the gene order in the substring.

**Table:** 

Single-point gene inversion:	Multi-point gene inversion:
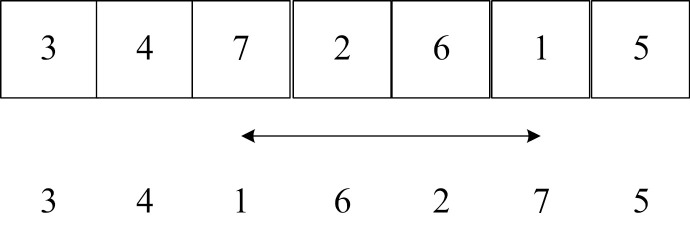	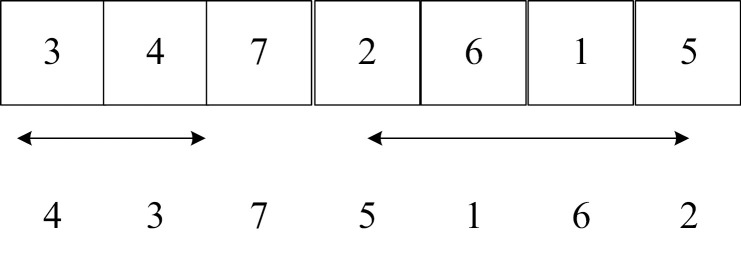

Step 6: Metropolis simulated annealing mechanism accept judgment. Metropolis can accept poor individuals with a certain probability, as shown in Eq (20).


$$\mathrm{\backslash [}P_{ij}=\left\{ \begin{array}{c} 1,\;f\left(j\right)\leq f\left(i\right)\\ e^{\left(\frac{f\left(i\right)-f\left(j\right)}{t}\right)},f\left(j\right)>f\left(i\right) \end{array}\right.\mathrm{\backslash ]}$$ (20)

Where, t is the annealing temperature, and the initial value of t is 90, the value of t decreases with the number of iterations.

Step 7: Determine whether the termination condition is met. If it is met, output the optimal solution, otherwise jump to Step 3.

### 4.2 Algorithm design in the dynamic adjustment stage

In order to ensure the running speed of the algorithm in the dynamic adjustment stage, the customers that have completed the distribution task and the customers that have cancelled the service demands in the optimal solution of the previous period are deleted, and the dynamic customer set $N_{2}$ is added as the first n chromosomes in the initial population in the dynamic adjustment stage. The remaining ( pop_size−n) chromosomes are generated by random generation, and the new population is decoded. The route is planned according to the surplus of agricultural products in the vehicles. When the surplus of agricultural products in the vehicles in transit to perform the distribution task cannot cover the customers’ demands, a new vehicle will be dispatched from the distribution center, completing the decoding process.

## 5 Implementation and result analysis

Firstly, based on the standard MDVRP example [48], a test data set is generated according to the problem characteristics, and the algorithm proposed in this paper is compared with two heuristic algorithms to analyze the performance of the algorithm. Secondly, the distribution network of three enterprises in Shanghai is used as an example to analyze and verify the effectiveness of the joint distribution strategy and two-stage dynamic optimization model proposed in this paper. All case experiments are programmed in MATLAB R2023b and run on a PC with an Intel i5 1.20GHz 1.50 GHz CPU.

### 5.1 PGHSAA Algorithm effectiveness analysis

In order to verify the effectiveness of the PGHSAA proposed in this paper, the PGHSAA algorithm is compared with the Grey Wolf Algorithm (GWO) and the Whale Algorithm (MOA). Both GWO and MOA are swarm intelligence optimization algorithms with strong convergence ability and less dependence on manually set parameters. The parameter settings of the three algorithms are shown in Table 3.

**Table 3:** GWO and MOA parameter setting.

Algorithm	Parameter setting
PHGSAA	popsize=200; iterations=500; $P_{c}=0.8$; $P_{m}=0.1$
GWO	popsize=200; iterations=500; α=2(1−iter/iterations); P=0.5; b=1
MOA	popsize=200; iterations=500; $c=2\exp\left[-{\left(4iter/iterations\right)}^{2}\right]$

This paper uses the data of distribution centers, vehicles, and customers’ coordinates in the MDVRP example as the basis for the experimental data in this section. Meanwhile, the service relationship between the distribution center and the customer is set, and the customers are assigned to each distribution center on average according to the number of distribution centers in different examples. The relevant data characteristics are detailed in Table 4.

**Table 4:** Example data characteristics.

Instance	Num	Qk	D_C	Instance	Num	Qk	D_C
Pr1	48	200	4	Pr6	288	175	4
Pr2	96	195	4	Pr7	72	200	6
Pr3	144	190	4	Pr8	144	190	6
Pr4	192	185	4	Pr9	216	180	6
Pr5	240	180	4	Pr10	288	170	6

(1) Comparison of solution results of different algorithms

The solution results of PGHSAA are compared with those of GWO and WOA. The results are shown in Table 5.

**Table 5:** Solution results of different algorithms.

Instance	Num	D_C	GWO	MOA	PGHSAA	GAP^GWO^(%)	GAP^WOA^(%)
Pr1	48	4	2214.99	2499.02	2590.27	−11.37	−14.49
Pr2	96	4	3695.49	4346.44	4673.03	−14.98	−20.92
Pr3	144	4	5887.84	6645.90	6851.44	−11.41	−14.06
Pr4	192	4	7663.78	8586.69	8912.45	−10.75	−14.01
Pr5	240	4	10532.52	12610.64	12926.24	−16.48	−18.52
Pr6	288	4	12299.15	14721.00	15277.14	−−16.45	−19.49
Pr7	72	6	3295.82	3742.90	3449.70	−11.94	−4.46
Pr8	144	6	5885.15	7445.12	7376.41	−20.95	−20.22
AVG	–	–	6434.34	7574.71	7757.08	−14.29	−15.77

Table 5 shows the example name (Instance), customer scale (Num), number of distribution centers (D_C), the optimal solution value of each algorithm and the difference value $GAP^{Y}$ between them. $GAP^{Y}=\left(PGHSAA-Y\right)/Y\;\times 100\;\%$, where *Y* represents GWO/WOA. As shown in Table 5, compared with GWO and WOA, PGHSAA has the best solution. The average total cost of 10 sets of data calculated by PGHSAA is 6434.34 yuan, which is 14.29% and 15.77% lower than that of GWO and WOA respectively. Therefore, the PGHSAA designed in this paper has better optimization ability and superiority than GWO and WOA algorithms when solving the optimization problem of agricultural products joint distribution network shared by vehicles and customers.

**Fig 8:**
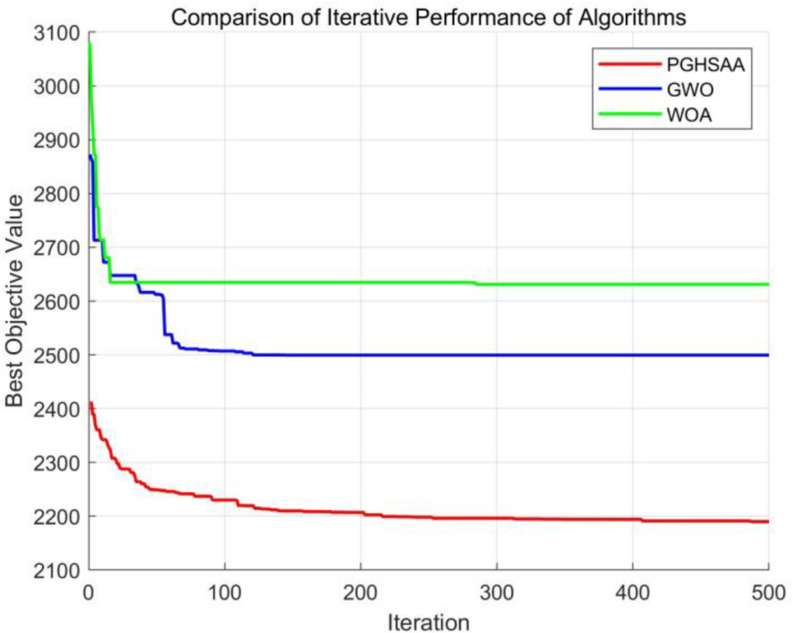
Comparative iteration diagram of different algorithms for solving Pr1.

In Fig 8, the PGHSAA algorithm has the following advantages over other algorithms when dealing with the joint distribution network optimization problem of agricultural products shared by vehicles and customers: first, the PGHSAA algorithm performs well in terms of the speed of convergence to the relative optimal solution. Second, the quality of the optimal solution it finds is relatively higher, which means that the PGHSAA algorithm has higher applicability and accuracy for solving the joint distribution network optimization problem of agricultural products under vehicles and customers sharing strategy.

**Fig 9:**
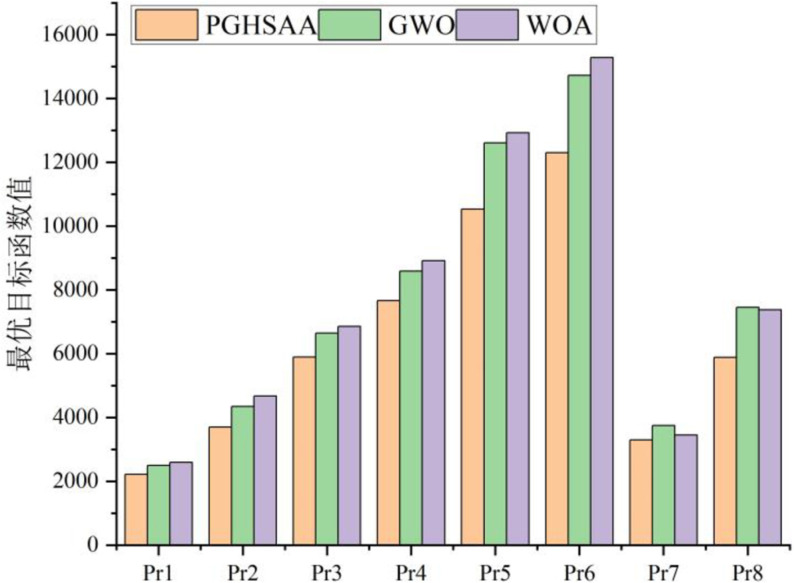
Comparison of the optimal objective function values obtained by three different algorithms under different scale test data sets.

For calculation examples of the same scale in the data set, the average value of the optimal objective function value is calculated, as shown in Fig 9. Experimental results show that the results obtained by the PGHSAA algorithm have significant advantages, with an average improvement of 14.29% compared to the GWO algorithm and an average improvement of 15.77% compared to the MOA algorithm.

(2) Stability analysis

**Fig 10:**
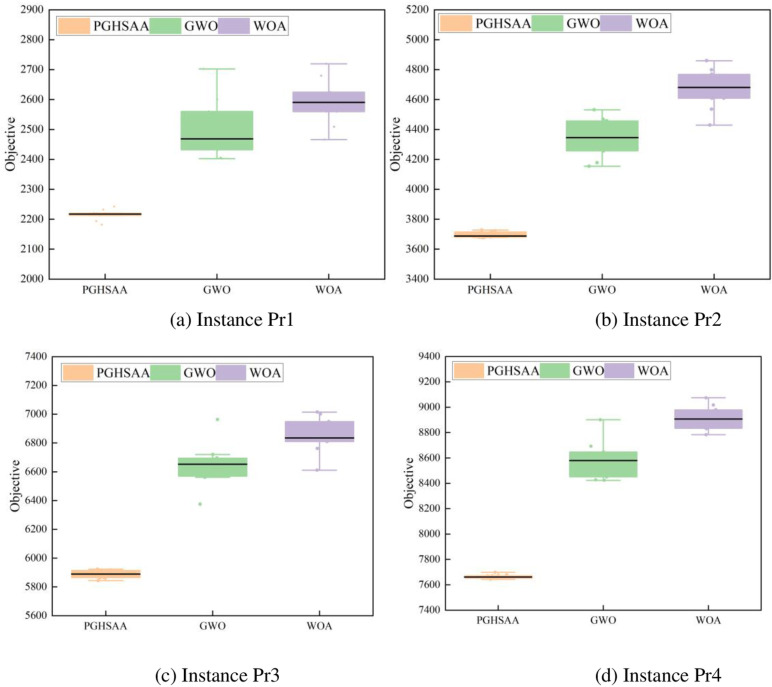
Distribution of objective function values solved by three algorithms. (a) Instance Pr1,(b) Instance Pr2, (c) Instance Pr3, (d) Instance Pr4.

In order to further analyze the solution stability of the three algorithms at different scales, four examples of Pr1, Pr2, Pr3, and Pr4 were selected, and box plots of the three algorithms for the objective function 10 times were drawn, as shown in Fig 10. In these four examples, the data distribution of the solution results obtained by the GWO and WOA algorithms is relatively discrete, and the difference between the data is relatively large. Taking Pr4 as an example, the variances of the GWO and WOA algorithms are 21319.73 and 9080.14 respectively, while the variance of the PGHSAA algorithm is 286.40, and the median of PGHSAA is always lower than the other two algorithms in the four examples, indicating that the PGHSAA algorithm is more reliable and has better robustness than the other two algorithms. In particular, as the problem scale increases, the solution advantage of the PGHSAA algorithm over the other two algorithms becomes more obvious.

**Fig 11:**
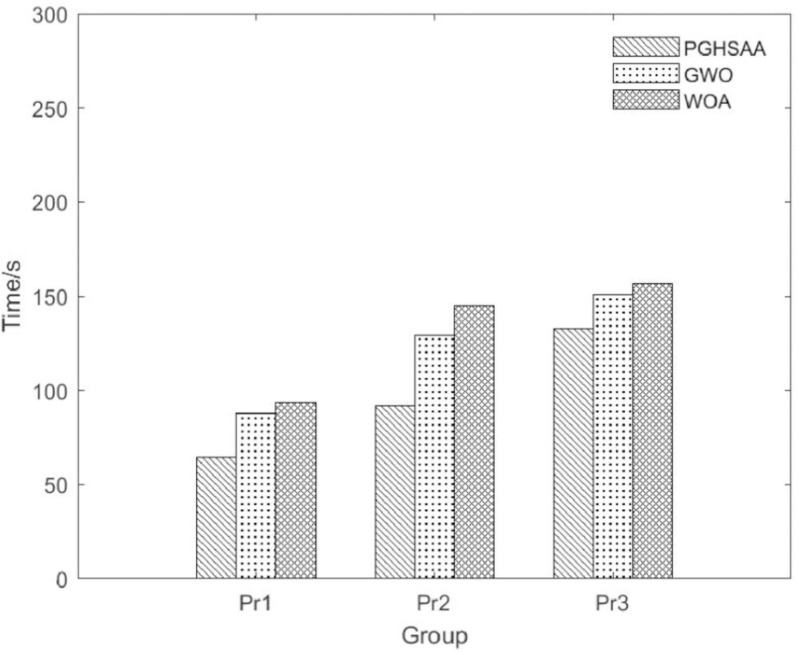
Algorithms runtime comparison.

To compare the computational efficiency of the three algorithms, this paper starts from the algorithm running time. As shown in Fig 11, in the Pr2 example (when the number of customers is 72), the running time of the PGHSAA algorithm has a significant advantage over the other two algorithms, which is only 91.84s. As the customer scale continues to increase, the running time of the three algorithms has increased significantly, but the running time of PGHSAA is always less than the other two algorithms.

### 5.2 Analysis of the effectiveness of distribution strategy

#### 5.2.1 Speed analysis during delivery.

**Fig 12:**
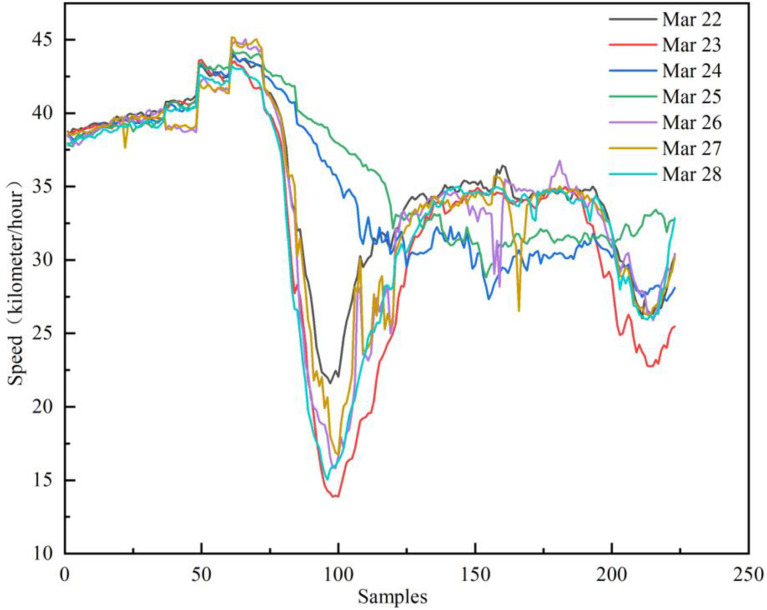
Map of Shanghai’s one week road network.

This paper selects the distribution center and customers’ information of three enterprises in *Shanghai*, China as analyzed example, and predicts the vehicle speed in Shanghai. First, Python is used to call Baidu Map API to crawl the average vehicle speed in Shanghai from 6 am to 8 pm every day from March 22 to March 28, and the line graph is shown in Fig 12.

As shown in Fig 12, the morning and evening peaks are more obvious on working days. The morning peak occurs at about 7:00 ~ 8:30, and the evening peak occurs between 17:30 ~ 18:30. The average speed of the road network during the morning and evening peaks is about 15km/h, which is in sharp contrast to the driving speed during non-morning and evening peaks. The roads are relatively smooth and the speed can reach 35km/h. This shows that it is very necessary for vehicles to consider the changes in driving speed at different times in the road network when performing delivery tasks. In addition, the evening peak on Friday lasts longer than the evening peak on other working days, and the driving speed during the corresponding period is also significantly lower than that on other working days, which is in line with the actual situation. Compared with Sunday and working days, the speed fluctuation range on Saturday is smaller.

**Fig 13:**
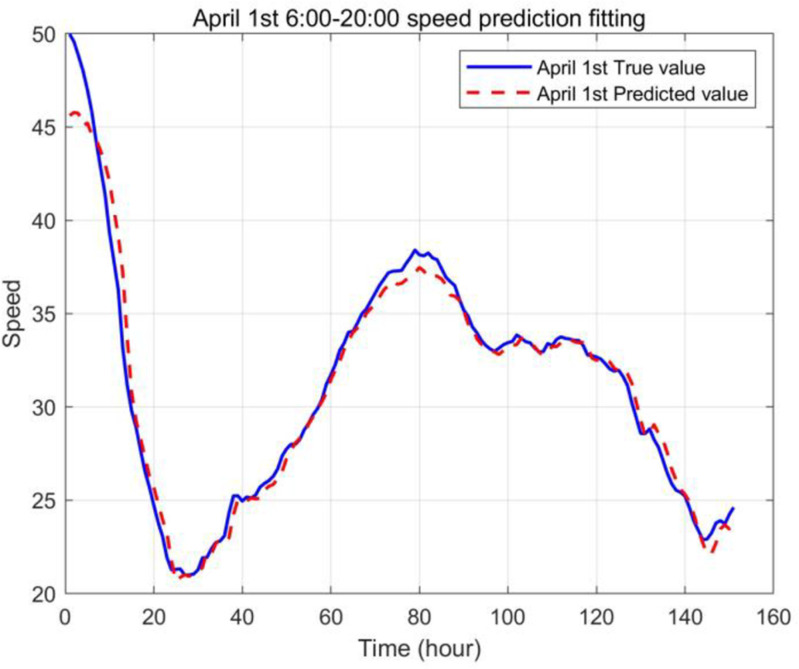
Fitting chart of actual and predicted road network speeds on April 1st.

The input sample data is the data of the road network speed in Shanghai from March 22 to April 1. The neural network is trained using the speed from March 29 to March 31, and the neural network is tested using the data on April 1. Fig 13 is a comparison chart of the actual value and predicted value of the road network speed, and the fitting effect is good.

The GA-BP prediction model established above can be used to predict the future driving speed of the road network. Taking the driving speed at 6:00 am on April 2 as an example, the input parameters of BP are the speed of the five time periods before 6:00 am on April 2 and the driving speed at 6:00 am on March 26, March 31 and April 1, and the driving speed at 6:00 am on April 2 is predicted.

#### 5.2.2 Optimization results and analysis.

In order to verify the effectiveness of the joint distribution strategy proposed in this paper, the distribution network of three enterprises in Shanghai is analyzed as an example. The information of the candidate distribution centers and customers is shown in S1 File. The start working time of the distribution center is 6 am and the ending working time is 8 pm. Based on the existing literature and multiple experimental calculations, other relevant parameter settings are shown in Table 6.

**Table 6:** Experimental related parameter values.

Parameter	Description	Value	Unit
$Q_{k}$	Capacity of delivery vehicles	4	ton
$F_{R}$	Fixed cost of setting up alternative distribution centers as joint distribution centers	500	yuan
$F_{K}$	Fixed cost of starting a vehicle once	100	yuan
$F_{d}$	Vehicle’s driving cost per unit time	25.2	yuan/hour
$F_{S}$	Unit cost of agricultural products damage	50	yuan/ton
$\varphi_{1}$	Waiting penalty cost per unit time	15	yuan/hour
$\varphi_{2}$	Cost of penalty for lateness per unit time	20	yuan/hour
$\beta_{1}$	The damage coefficient of agricultural products during distribution	0.001	/
$\beta_{2}$	Damage coefficient of agricultural products during loading and unloading	0.003	/

**Fig 14:**
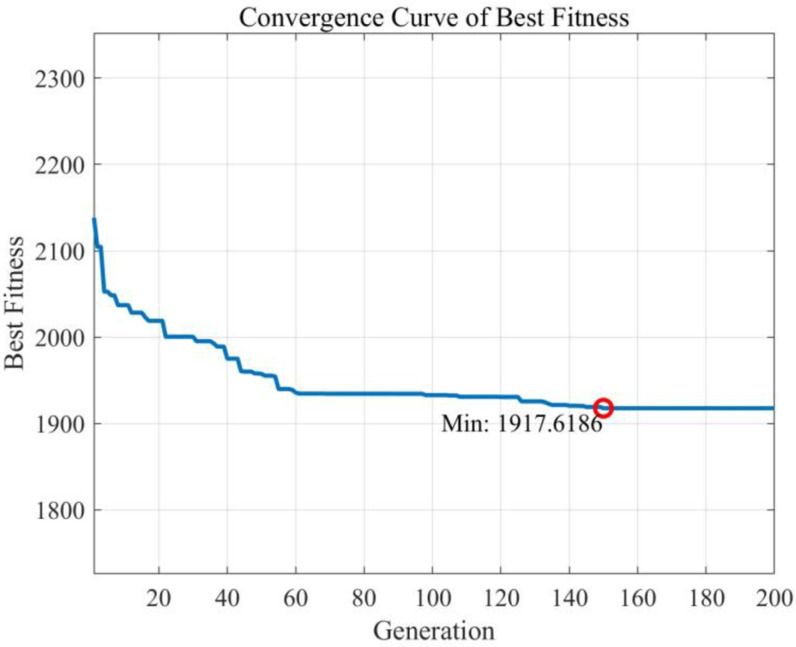
Pre-optimization iteration process.

**Fig 15:**
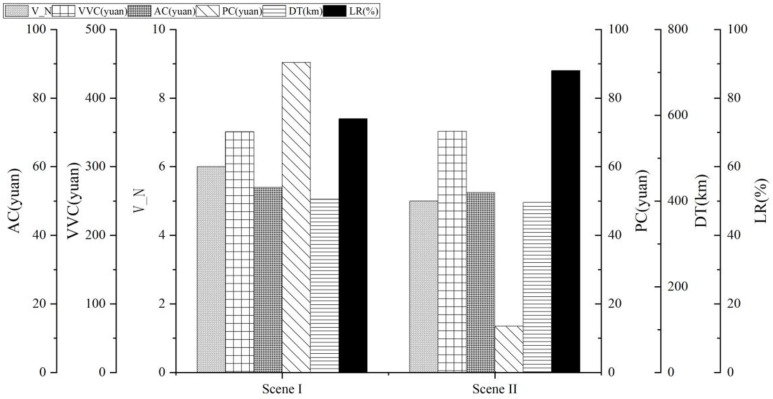
Comparison of indicators before and after optimization of agricultural products joint distribution network.

With the goal of minimizing the total cost including the distribution center operating cost, vehicle fixed cost, vehicle driving cost, agricultural products loss cost and time window penalty cost, the PGHSAA algorithm is applied to optimize the agricultural products joint distribution network under vehicles and customers sharing strategy, and the distribution plan for the pre-optimization stage is obtained. The algorithm iteration process is shown in Fig 14, and the comparison results of various costs before and after optimization are shown in Table 7 and Fig 15.

**Table 7:** Comparison of Results Before and After Optimization of Agricultural Product Joint Distribution Network.

Scene	V_N	VVC(yuan)	AC(yuan)	PC(yuan)	DD(km)	LR(%)
Scene I	6	350.88	53.95	90.39	404.10	0.74
Scene II	5	351.74	52.42	13.46	396.74	0.88

Table 7 shows vehicle number (V_N), driving distance (DD) and loading rate (LR). It can be seen from Table 7 and Fig 15 that under vehicles and the customers sharing strategy, the vehicle driving cost and vehicle loading rate increased by 0.24% and 18.92% respectively; the number of distribution vehicles used has been saved by 16.67%, the agricultural products loss cost has been reduced by 2.84%. Compared with traditional independent distribution, the time window penalty cost under joint distribution has been greatly reduced, saving 85.11%, and the driving distance has been reduced by 1.82%.

### 5.3 Dynamic model validity analysis

When the vehicle is performing the delivery task, all the dynamic event information received by the distribution center is shown in Table 8. Table 8 shows the number of dynamic events (S_Num), the number of customer (C_N).

**Table 8:** Dynamic events information.

S_Num	C_N	X/km	Y/km	Dynamic Events	Dynamic Type
1	16	26.225082	1.212637298	6:33	Order Cancellation
2	32	11.410467	10.51628029	6:39	New Customers (0.3t)
3	12	24.160815	4.504523993	7:36	Increased demand (0.2t)
4	33	17.026401	12.57667489	7:40	New Customers (0.4t)
5	34	25.101318	4.377762163	8:32	New Customers (0.7t)

The dynamic events occurred in the three dynamic service time windows of [6:33,7:00], [7:30,8:00], and [8:30,9:00]. At 7:00, the information of the delivery vehicle is shown in Table 9.

**Table 9:** Distribution vehicle information at 9.

Vehicle	Vehicle capacity	Virtual node	Remaining capacity/t
Vehicle 1	4t	14	1.4
Vehicle 2	4t	11	1.4
Vehicle 3	4t	25	2.8
Vehicle 4	4t	17	1.4
Vehicle 5	4t	20	2

As shown in Table 9, at 7:00, with customers 14, 11, 25, 17 and 20 as virtual nodes, the number of vehicle configurations and the driving routes are adjusted. After multiple experiments, it is known that a satisfactory solution can be obtained after about 86 iterations. The maximum number of evolution generations in this stage is set to 200. The average time for the program to run ten times is 11.80 seconds, which is feasible for dynamic adjustment. After adjustment, the departure time of the vehicle from the virtual node is 7:28, 7:38, 8:04, 7:44, and 7:28. The specific plan of the adjusted delivery route is shown in Table 10. The data in [] in Table 10 is the adjusted vehicle delivery route.

**Table 10:** Adjusted delivery plan at 7:00.

Vehicle	Vehicle routing	DD	Cost/yuan	Total cost/yuan	Response time/s
Vehicle 1	D3-4-7-6-[14-21-32-D3]	87.93	276.72	1378.36	11.80
Vehicle 2	D3-10–9-[11-12-28-D3]	89.67	276.52		
Vehicle 3	D3-3–1-[25-19-23-26-24-D3]	90.42	288.63		
Vehicle 4	D3-5-2-18-[17-15-27-D3]	79.19	275.62		
Vehicle 5	D3-8–13-[20–22-D3]	68.89	260.87		

At 8:00, the distribution plan is re-planned with customers 14, 12, 25, 15 and 20 as virtual nodes. The departure times of the vehicles from the virtual nodes after adjustment are 8:28, 8:38, 8:03, 8:44 and 8:29. The specific plan is shown in Table 11. The data in [] in Table 11 are the adjusted vehicle distribution routes.

**Table 11:** Adjusted delivery plan at 8:00.

Vehicle	Vehicle routing	DD	Cost/yuan	Total cost/yuan	Response time/s
Vehicle 1	D3-4-7-6-[14-32-33-28-D3]	88.25	288.17	1403.26	9.49
Vehicle 2	D3-10-9-11-[12–19-D3]	93.25	278.78		
Vehicle 3	D3-3–1-[25-23-26-24-D3]	90.42	288.63		
Vehicle 4	D3-5-2-18-17-[15–21-D3]	80.07	279.22		
Vehicle 5	D3-8–13-[20-22-27-D3]	71.78	268.47		

Dynamic event 5 occurs in [8:30,9:00]. According to the distribution plan, customers 33, 23 and 21 are used as virtual nodes. The demand of customer 34 is processed immediately and the subsequent distribution path is adjusted. The specific distribution plan is shown in Table 12.

**Table 12:** Adjusted delivery plan at 8:32.

Vehicle	Vehicle routing	DD	Cost/yuan	Total cost/yuan	Response time/s
Vehicle 1	D3-4-7-6-14-32-[33–28-D3]	88.25	288.17	1414.68	5.28
Vehicle 2	D3-10-9-11-12-34-D3	98.66	290.19		
Vehicle 3	D3-3-1-25-19-[23-26-24-D3]	90.42	288.63		
Vehicle 4	D3-5-2-18-17-15-[21-D3]	80.07	279.22		
Vehicle 5	D3-8-13-20-22-27-D3	71.78	268.47		

To further verify the superiority of the idea of responding to new customers proposed in this paper, the idea of scheduled batch processing and the idea of combining immediate processing and scheduled batch processing proposed in this paper are used to adjust the distribution route of vehicles, and the optimal solution is selected to compare and analyze the indicators such as the number of vehicles, vehicle driving distance and total cost. The comparison results are shown in Table 13.

**Table 13:** Comparison results.

Processing means	Scheduled batch processing	Integrating immediate processing and scheduled batch processing in this paper
Optimal distribution scheme	D3-4-7-6-33-32-20-D3 D3-10-9-11-15-14-27-D3 D3-3-1-25-19-22-24-D3 D3-5-2-18-17-26-23-D3 D3-8-13-12-21-D3 D3-34-D3	D3-4-7-6-14-32-[33–28-D3] D3-10-9-11-12-17-34-D3 D3-3-1-25-19-[23-26-24-D3] D3-5-2-18-17-15-[21-D3] D3-8-13-20-22-27-D3
Vehicle number	6	5
Vehicle driving distance	466.55	429.18
Total cost	1661.63	1414.68

It can be seen from Table 13 that the number of vehicles invoked by the combination of immediate processing and scheduled batch processing and the idea of scheduled batch processing only used in this paper is reduced by 16.70%, and the driving distance and total cost of the optimization scheme are effectively reduced. Compared with scheduled batch processing, the driving distance of vehicles is reduced by 8.01%, and the total cost is reduced by 14.86%, and the response ability to dynamic events is improved.

## 6 Conclusion

### 6.1 Main conclusions

In order to solve the challenges in the actual distribution of agricultural products, such as the delayed response of the distribution network caused by the change of customer demands and the dynamics of the traffic network, and the high distribution cost caused by the independent distribution of enterprises, this paper first predicts the vehicle speed from the perspective of data-driven. On this basis, in order to minimize the total distribution cost, a two-stage dynamic optimization model of pre-optimization and dynamic adjustment of agricultural products joint distribution network under vehicles and customers sharing strategy is established to improve the distribution efficiency of agricultural products. In order to effectively deal with this complex problem, the disruption event processing idea of combining immediate processing with scheduled batch processing and the PGHSAA are proposed.

Through multiple sets of examples and case analysis, the following conclusions are drawn:

First, the PGHSAA shows obvious advantages in solving the optimization problem of agricultural products joint distribution network. It converges quickly, and the quality of the solution sought is high, with certain robustness.
Second, compared with the traditional independent distribution strategy, the joint distribution strategy shared by vehicles and customers completes the distribution of agricultural products, which increases the vehicle loading rate by 18.92%, saves a certain number of distribution vehicles, and greatly reduces the time window penalty cost.
Third, in response to changes in customer demands during the delivery process, the immediate processing and scheduled batch processing ideas proposed in this paper can take into account both customer requests and the computational complexity of the algorithm, and effectively respond to customer demands.

### 6.2 Implications

In summary, logistics service providers should actively promote the digitalization, intelligence and sharing of agricultural products distribution networks, improve the efficiency of agricultural products distribution and reduce distribution costs. Secondly, by combining immediate and scheduled batch processing ideas, it is possible to effectively respond to dynamic customers arriving during the distribution process and changes in the demands of original customers, and improve customer satisfaction and gain market advantages. This paper expands on the theory of joint distribution, aiming to enrich the research in the field of joint distribution, and the research conclusions obtained can provide a certain reference for future agricultural products joint distribution network optimization decision-making.

### 6.3 Limitation and future research

With the continuous development of artificial intelligence technology, reinforcement learning technology has strong learning ability and fast calculation speed. The trained reinforcement learning model can optimize the agricultural products distribution network in real time online. Therefore, online intelligent optimization of agricultural products distribution network based on reinforcement learning technology is a topic worthy of in-depth research in the future. In addition, due to the lack of virtual scenes that iteratively interact with the actual distribution process, the feasibility and accuracy of the solution cannot be guaranteed. Digital twin technology is a feasible idea to alleviate the above problems. It provides experimental environment support for the training and verification of agricultural products distribution network optimization models through high-fidelity simulation models that are consistent with real distribution environment elements (such as traffic environment, distribution vehicles, etc.), which can effectively alleviate the difficulty of deploying models from simulation to reality (sim to real). Therefore, digital twin technology can also be used as one of the future research directions.

## Supporting information

S1 File The information of the candidate distribution centers and customers of Section 5.2.2 is shown in S1 File.
(RAR)

## References

[1] China International Capital Corporation Credit Consulting Center. Analysis and Forecast of the Current Status and Future Development Prospects of China’s Agricultural Products Logistics Industry. 2023. Available from: https://caifuhao.eastmoney.com/news/20231229110647971532520

[2] Yadav VS, Singh AR, Raut RD, Cheikhrouhou N. Design of multi-objective sustainable food distribution network in the Indian context with multiple delivery channels. Computers & Industrial Engineering. 2021;160:107549. doi: 10.1016/j.cie.2021.107549

[3] Chen J, Dan B, Shi J. A variable neighborhood search approach for the multi-compartment vehicle routing problem with time windows considering carbon emission. Journal of Cleaner Production. 2020;277:123932. doi: 10.1016/j.jclepro.2020.123932

[4] Yıldırım UM, Çatay B. An enhanced network-consistent travel speed generation scheme on time-dependent shortest path and routing problems. IEEE Transactions on Intelligent Transportation Systems. 2020;23(2):873–884.

[5] Choi J, Do Chung B. Optimizing vehicle route, schedule, and platoon formation considering time-dependent traffic congestion. Computers & Industrial Engineering. 2024;192:110205. doi: 10.1016/j.cie.2024.110205

[6] Wang XH, Duan YJ. Mechanisms and path for lowering logistics cost in the whole society driven by new quality productive forces. China business and market. 2024;38(7):15–24.

[7] Zhu F, Lv Y, Chen Y, Wang X, Xiong G, Wang F-Y. Parallel Transportation Systems: Toward IoT-Enabled Smart Urban Traffic Control and Management. IEEE Trans Intell Transport Syst. 2020;21(10):4063–4071. doi: 10.1109/tits.2019.2934991

[8] Vivekananda GN, Jarwar MA, Jaber MM, Prakash C, Buddhi D, Gnanasigamani LJ, Sanz-Prieto I. Effective two-tier tokenization for intelligent transportation supply chain systems using hybrid optimized query expansion. Multimedia Tools and Applications. 2022;21:1–29.

[9] Ren X, Fan H, Ma M, Fan H, Yue L. Time-dependent hydrogen fuel cell vehicle routing problem with drones and variable drone speeds. Computers & Industrial Engineering. 2024;193:110330. doi: 10.1016/j.cie.2024.110330

[10] Huang Y, Zhang J. Solving vehicle routing problem using deep reinforcement learning. Journal of Transportation Engineering and Information. 2022;20(3):114–127.

[11] Liu SY, Chen T’E, Chen D, Zhang C, Wang C. Time-varying heterotypic-vehicle cold chain logistics distribution path optimization model. Smart Agriculture. 2021;3(3): 139–151.

[12] Wu D, Yan R, Jin H, Cai F. An Adaptive Nutcracker Optimization Approach for Distribution of Fresh Agricultural Products with Dynamic Demands. Agriculture. 2023;13(7):1430. doi: 10.3390/agriculture13071430

[13] Ma YF, Ying B, Zhou XY. Multi-agent optimization model and algorithm for perishable food location-routing problem with conflict and coordination. System Engineering – Theory & Practice. 2020;40(12):3194–3209.

[14] Govindan K, Jafarian A, Khodaverdi R, Devika K. Two-echelon multiple-vehicle location–routing problem with time windows for optimization of sustainable supply chain network of perishable food. International Journal of Production Economics. 2014;152:9–28. doi: 10.1016/j.ijpe.2013.12.028

[15] Rahmanifar G, Mohammadi M, Golabian M, Sherafat A, Hajiaghaei-Keshteli M, Fusco G, et al. Integrated location and routing for cold chain logistics networks with heterogeneous customer demand. Journal of Industrial Information Integration. 2024;38:100573. doi: 10.1016/j.jii.2024.100573

[16] Liu H, Zhang J, Zhou Z, Dai Y, Qin L. A Deep Reinforcement Learning-Based Algorithm for Multi-Objective Agricultural Site Selection and Logistics Optimization Problem. Applied Sciences. 2024;14(18):8479. doi: 10.3390/app14188479

[17] Zhou X, Li J, Xie F, Fang J. Research on origin-based cold storage location and routing optimization of fresh agricultural products based on hybrid whale algorithm. Sci Rep. 2024;14(1):21078. doi: 10.1038/s41598-024-72170-z 39256605 PMC11387780

[18] Chen X, Jiu Y, Hu D. Two-Echelon Location–Routing Problem of Perishable Products Based on the Integrated Mode of In-Store Pick-Up And Delivery. Transportation Research Record: Journal of the Transportation Research Board. 2024;2678(8):622–636. doi: 10.1177/03611981231218008

[19] Wang S, Tao F, Shi Y. Optimization of Location-Routing Problem for Cold Chain Logistics Considering Carbon Footprint. Int J Environ Res Public Health. 2018;15(1):86. doi: 10.3390/ijerph15010086 29316639 PMC5800185

[20] Navazi F, Sazvar Z, Tavakkoli-Moghaddam R. A sustainable closed-loop location-routing-inventory problem for perishable products. Scientia Iranica. 2023;30(2):757–783.

[21] Wang NM, Liang XY, Zhang M, He ZW. Vehicle routing problem of multi-trips for perishable product delivery with considering individual customer satisfaction. Operation Research and Management Science, 2024;33(4):14–20.

[22] Psaraftis. Vehicle routing: methods and studies. Amsterdam: Elsevier Science Publishers; 1988, 223–248.

[23] Luo L, Chen HX, Wu Z, Xie XL, Liu CS. Vehicle routes of cold chain distribution for fresh agricultural product based on the dual functions of the traffic and weather conditions. Systems Engineering. 2022;40(6):67–75.

[24] Liu CS, Shen LZ, Sheng HY, Lyu XY, Qu YP. Research on low-carbon time-dependent vehicle routing problem with traffic congestion avoidance approaches. Control and Decision. 2020;35(10):2486–2496.

[25] Fu ZH, Liu CS. Research on time-dependent vehicle routing problem of fresh food e-commerce distribution. Computer Engineering and Applications. 2021;57(1):271–278.

[26] Li Y, Fan HM, Zhang XN. A Periodic Optimization Model and Solution for Capacitated Vehicle Routing Problem with Dynamic Requests. Chinese Journal of Management Science. 2022;30(8):254–266.

[27] Fan HM, Zhang YG, Tian PJ, Ren XX. Dynamic vehicle routing problem of heterogeneous fleets with time-dependent networks. Systems engineering-Theory & Practice. 2022;42(2):455–470.

[28] Ding QL, Hu XP, Wei J, Yang J. Research on multi-objective optimization model and algorithm of cold storage multi-temperature joint delivery under dynamic demand. Operations Research and Management Science. 2021;30(12):13–19.

[29] Ding QL, Jiang Y, Wang WJ, Qi F. Disruption management model for cold chain delivery of fresh agricultural products. Systems engineering-Theory & Practice. 2017;37(9):2320–2330.

[30] Shi XL. The development trend of agricultural products logistics and the countermeasures. China business and market. 2015;29(7):25–29.

[31] Li JJ, Xu JW. Practical dilemma and implementation approaches for reducing costs in China’s agricultural product logistics. China business and market. 2024;38(10):33–44.

[32] Paul J, Agatz N, Spliet R, Koster RD. Shared Capacity Routing Problem − An omni-channel retail study. European Journal of Operational Research. 2019;273(2):731–739. doi: 10.1016/j.ejor.2018.08.027

[33] Wang Y, Zhe J, Wang X, Sun Y, Wang H. Collaborative Multidepot Vehicle Routing Problem with Dynamic Customer Demands and Time Windows. Sustainability. 2022;14(11):6709. doi: 10.3390/su14116709

[34] Zhang J, Li YF. Location-routing problem of fresh product distribution in epidemic environment. Chinese Journal of Management Science. Forthcoming.

[35] Liu JL, Ma ZJ. Multi-depot open vehicle routing problem with time windows based on vehicle leasing and sharing. Systems engineering-Theory & Practice. 2013;33(3):666–675.

[36] Wang Y, Zhang J, Liu Y, Xu MZ. Optimization of fresh goods multi-center vehicle routing problem based on resource sharing. Chinese Journal of Management Science. 2022;30(11):272–285.

[37] Xia YK, Deng YD, Pang Y, Wang ZW, Gao L. Fresh food vehicle routing problem with split deliveries and customer classification. Computer Integrated Manufacturing Systems. 2021;27(4):1238–1248.

[38] Dai HY, Tao JW, Jiang H, Zhou WH. Paradigm shift for big data-driven decision making: New paradigm for O2O on-demand logistics. Journal of Management Sciences in China. 2023;26(5):53–69.

[39] Sun HJ, Yang S, Lv Y, Gao ZY. Research on data-driven bi-level game problems in resource allocation of sharing mobility systems. Journal of Management World. 2023;39(4):160–174.

[40] Xiong H, Yan HL. Research on the data-driven mechanism of intelligent order dispatching of takeaway platform. Nankai Business Review. 2022;25(2):15–23.

[41] Li XC, Li MZ, Zeng QC, Yang A. Two-layer scheduling model of quay cranes and automated guided vehicles with data-driven methods at automated container terminals. Frontiers of Science and Technology of Engineering Management. Forthcoming.

[42] Du M, Sai A, Kong N. A data-driven optimization approach for multi-period resource allocation in cholera outbreak control. European Journal of Operational Research. 2021;291(3):1106–1116. doi: 10.1016/j.ejor.2020.09.052

[43] Sarkar S, Pramanik A, Maiti J, Reniers G. COVID-19 outbreak: A data-driven optimization model for allocation of patients. Comput Ind Eng. 2021;161:107675. doi: 10.1016/j.cie.2021.107675 34522063 PMC8428993

[44] Chu H, Zhang W, Bai P, Chen Y. Data-driven optimization for last-mile delivery. Complex Intell Syst. 2021;9(3):2271–2284. doi: 10.1007/s40747-021-00293-1

[45] Ma CX, Xue FS, Ma CR, Li HJ. Route optimization of fresh food distribution under time-varying network and hybrid adjustment strategy. Journal of transportation systems engineering and information technology. 2023;23(4):298–306.

[46] Yan F, Peng TT, Shen CR. Time-space cluster based location-routing problem with capacitate constraints. Control and Decision. 2021;36(10):2504–2510.

[47] Zhang HZ, Liu Y, Ni J. A partheno-genetic hybrid ant colony algorithm for solving the MOVRPFTW based on customer satisfaction. Journal of Systems & Management. 2019;28(5):927–933.

[48] Fu ZH, Liu CS. Research on multi-depot vehicle routing problem based on joint distribution mode. Computer Engineering and Applications. 2021;57(16):291–298.

